# Supplementary material for: Modeling glioblastoma heterogeneity as a dynamic network of cell states
Source: Mol Syst Biol. 2021 Sep 16;17(9):e10105. doi: 10.15252/msb.202010105 (PMC8444284; doi:10.15252/msb.202010105)
Supplement: Supplementary file 6 — Source Data for Figure 5 [file MSB-17-e10105-s004.zip › Figure5A_sourcedata/GSEA_3071/hallmarks_stateII.GseaPreranked.1623742536692/HALLMARK_MYOGENESIS.html]

Details for gene set HALLMARK\_MYOGENESIS[GSEA]

|  || Dataset | state23071 |
| Phenotype | NoPhenotypeAvailable |
| Upregulated in class | na\_neg |
| GeneSet | HALLMARK\_MYOGENESIS |
| Enrichment Score (ES) | -0.3319506 |
| Normalized Enrichment Score (NES) | -1.6809381 |
| Nominal p-value | 0.0073394496 |
| FDR q-value | 0.11637386 |
| FWER p-Value | 0.356 |
Table: GSEA Results Summary

  

Fig 1: Enrichment plot: HALLMARK\_MYOGENESIS      
 Profile of the Running ES Score & Positions of GeneSet Members on the Rank Ordered List

  

| PROBE | GENE SYMBOL | GENE\_TITLE | RANK IN GENE LIST | RANK METRIC SCORE | RUNNING ES | CORE ENRICHMENT || 1 | TAGLN |  |  | 43 | 1.279 | 0.0192 | No |
| 2 | GADD45B |  |  | 146 | 0.965 | 0.0165 | No |
| 3 | TPM2 |  |  | 150 | 0.957 | 0.0381 | No |
| 4 | TEAD4 |  |  | 206 | 0.869 | 0.0448 | No |
| 5 | FST |  |  | 226 | 0.849 | 0.0600 | No |
| 6 | BIN1 |  |  | 243 | 0.830 | 0.0754 | No |
| 7 | COL4A2 |  |  | 264 | 0.795 | 0.0890 | No |
| 8 | COL6A3 |  |  | 291 | 0.768 | 0.1005 | No |
| 9 | SMTN |  |  | 292 | 0.766 | 0.1184 | No |
| 10 | COL1A1 |  |  | 294 | 0.761 | 0.1359 | No |
| 11 | MYH9 |  |  | 323 | 0.735 | 0.1462 | No |
| 12 | MYO1C |  |  | 490 | 0.632 | 0.1198 | No |
| 13 | SPHK1 |  |  | 551 | 0.603 | 0.1191 | No |
| 14 | PDLIM7 |  |  | 580 | 0.587 | 0.1258 | No |
| 15 | NQO1 |  |  | 732 | 0.522 | 0.1006 | No |
| 16 | TPM3 |  |  | 887 | 0.473 | 0.0736 | No |
| 17 | HBEGF |  |  | 972 | 0.451 | 0.0633 | No |
| 18 | COL6A2 |  |  | 1021 | 0.437 | 0.0616 | No |
| 19 | SSPN |  |  | 1081 | 0.421 | 0.0569 | No |
| 20 | CDKN1A |  |  | 1088 | 0.419 | 0.0652 | No |
| 21 | KLF5 |  |  | 1294 | 0.380 | 0.0233 | No |
| 22 | FDPS |  |  | 1348 | 0.367 | 0.0187 | No |
| 23 | FGF2 |  |  | 1679 | 0.313 | -0.0557 | No |
| 24 | KIFC3 |  |  | 1764 | 0.303 | -0.0694 | No |
| 25 | COL15A1 |  |  | 1798 | 0.299 | -0.0706 | No |
| 26 | EFS |  |  | 2326 | -0.262 | -0.1950 | No |
| 27 | DTNA |  |  | 2414 | -0.272 | -0.2102 | No |
| 28 | AK1 |  |  | 2446 | -0.276 | -0.2114 | No |
| 29 | MEF2A |  |  | 2523 | -0.289 | -0.2235 | No |
| 30 | RIT1 |  |  | 2594 | -0.300 | -0.2338 | No |
| 31 | KCNH2 |  |  | 2610 | -0.302 | -0.2304 | No |
| 32 | SORBS1 |  |  | 2639 | -0.307 | -0.2302 | No |
| 33 | HDAC5 |  |  | 2651 | -0.308 | -0.2257 | No |
| 34 | MAPRE3 |  |  | 2863 | -0.346 | -0.2699 | No |
| 35 | ATP6AP1 |  |  | 3010 | -0.377 | -0.2973 | No |
| 36 | AEBP1 |  |  | 3026 | -0.379 | -0.2921 | No |
| 37 | NAV2 |  |  | 3083 | -0.393 | -0.2968 | No |
| 38 | AGL |  |  | 3134 | -0.410 | -0.2996 | No |
| 39 | NOTCH1 |  |  | 3202 | -0.429 | -0.3062 | No |
| 40 | EIF4A2 |  |  | 3289 | -0.453 | -0.3169 | No |
| 41 | MEF2C |  |  | 3301 | -0.457 | -0.3089 | No |
| 42 | PRNP |  |  | 3383 | -0.491 | -0.3175 | No |
| 43 | CHRNB1 |  |  | 3424 | -0.511 | -0.3155 | No |
| 44 | GSN |  |  | 3487 | -0.537 | -0.3183 | Yes |
| 45 | SVIL |  |  | 3490 | -0.537 | -0.3063 | Yes |
| 46 | ERBB3 |  |  | 3561 | -0.578 | -0.3101 | Yes |
| 47 | MRAS |  |  | 3613 | -0.611 | -0.3085 | Yes |
| 48 | EPHB3 |  |  | 3619 | -0.616 | -0.2953 | Yes |
| 49 | GABARAPL2 |  |  | 3768 | -0.759 | -0.3142 | Yes |
| 50 | MYL6B |  |  | 3788 | -0.777 | -0.3008 | Yes |
| 51 | CHRNA1 |  |  | 3874 | -0.892 | -0.3010 | Yes |
| 52 | CDH13 |  |  | 3908 | -0.963 | -0.2867 | Yes |
| 53 | WWTR1 |  |  | 3936 | -1.027 | -0.2694 | Yes |
| 54 | PKIA |  |  | 3950 | -1.056 | -0.2479 | Yes |
| 55 | NCAM1 |  |  | 3966 | -1.106 | -0.2258 | Yes |
| 56 | APP |  |  | 3982 | -1.164 | -0.2023 | Yes |
| 57 | ITGA7 |  |  | 4030 | -1.454 | -0.1800 | Yes |
| 58 | IGFBP3 |  |  | 4058 | -1.726 | -0.1463 | Yes |
| 59 | CLU |  |  | 4069 | -1.908 | -0.1042 | Yes |
| 60 | CKB |  |  | 4075 | -2.149 | -0.0553 | Yes |
| 61 | CRYAB |  |  | 4088 | -2.598 | 0.0025 | Yes |
Table: GSEA details [plain text format]

  

Fig 2: HALLMARK\_MYOGENESIS: Random ES distribution      
 Gene set null distribution of ES for **HALLMARK\_MYOGENESIS**
